# Neuraxial anesthesia versus general anesthesia for urological surgery: systematic review

**DOI:** 10.1590/1516-3180.2013.1313535

**Published:** 2013-06-01

**Authors:** Fabiano Timbó Barbosa, Aldemar Araújo Castro

**Affiliations:** I MSc. Professor, Surgery Department, Universidade Federal de Alagoas, Maceió, Alagoas, Brazil.; II MSc. Professor, Surgery Department, Universidade Estadual de Ciências da Saúde de Alagoas, Maceió, Alagoas, Brazil.

**Keywords:** Mortality, Anesthesia, general, Anesthesia, epidural, Anesthesia, spinal, Review [publication type], Mortalidade, Anestesia geral, Anestesia epidural, Raquianestesia, Revisão

## Abstract

**CONTEXT AND OBJECTIVE:**

Choosing the best anesthetic technique for urological surgery with the aim of mortality reduction remains controversial. The objective here was to compare the effectiveness and safety of neuraxial anesthesia versus general anesthesia for urological surgery.

**DESIGN AND SETTING:**

Systematic review, Universidade Federal de Alagoas.

**METHODS:**

We searched the Cochrane Central Register of Controlled Trials in the Cochrane Library (Issue 10, 2012), Medline via PubMed (1966 to October 2012), Lilacs (1982 to October 2012), SciELO and EMBASE (1974 to October 2012). The reference lists of the studies included and of one systematic review in the same field were also analyzed. The studies included were randomized controlled trials (RCT) that analyzed neuraxial anesthesia and general anesthesia for urological surgery.

**RESULTS:**

The titles and abstracts of 2720 articles were analyzed. Among these, 16 studies were identified and 11 fulfilled the inclusion criteria. One RCT was published twice. The study validity was: Jadad score > 3 in one RCT; seven RCTs with unclear risk of bias as the most common response; and five RCTs not fulfilling half of the Delphi list items. The frequency of mortality was not significant between study groups in three RCTs. Meta-analysis was not performed.

**CONCLUSION:**

At the moment, the evidence available cannot prove that neuraxial anesthesia is more effective and safer than general anesthesia for urological surgery. There were insufficient data to pool the results relating to mortality, stroke, myocardial infarction, length of hospitalization, quality of life, degree of satisfaction, postoperative cognitive dysfunction and blood transfusion requirements.

## RESUMO

**CONTEXTO E OBJETIVO:**

A escolha da melhor técnica anestésica para cirurgias urológicas para reduzir mortalidade permanece controversa. O objetivo foi comparar a efetividade e segurança da anestesia neuroaxial versus anestesia geral para cirurgias urológicas.

**TIPO DE ESTUDO E LOCAL:**

Revisão sistemática, Universidade Federal de Alagoas.

**MÉTODO:**

Fizemos a busca em Cochrane Central Register of Controlled Trials na Cochrane Library (2012, volume 10), Medline via PubMed (1966 até outubro de 2012), Lilacs (1982 até outubro de 2012), SciELO e EMBASE (1974 até outubro de 2012). As listas de referências dos estudos incluídos e de uma revisão sistemática em urologia também foram analisadas. Os estudos incluídos foram ensaios clínicos randomizados (ECR) que analisaram as anestesias neuroaxial e geral para cirurgias urológicas.

**RESULTADOS:**

Os títulos e resumos de 2.720 artigos foram analisados. Entre eles, 16 estudos foram identificados e 11 preencheram os critérios de inclusão. Um ECR foi publicado duas vezes. A validade dos estudos foi: escore de Jadad > 3 em um ECR; sete ECRs com indeterminado risco de viés como a resposta mais comum; cinco ECR sem preenchimento da metade dos itens da lista de Delphi. A frequência de mortalidade não foi significante entre os grupos de estudo em três ECR. A metanálise não foi realizada.

**CONCLUSÃO:**

Até o momento, as evidências disponíveis não puderam provar que a anestesia neuroaxial seja mais efetiva e segura do que a anestesia geral para cirurgias urológicas. Não houve dados suficientes para reunir os resultados de mortalidade, infarto cerebral, infarto do miocárdio, duração de internação hospitalar, qualidade de vida, grau de satisfação, disfunção cognitiva pós-operatória e necessidade de transfusão sanguínea.

## INTRODUCTION

Choosing the best anesthetic technique for urological surgery with the aim of mortality reduction remains controversial.^1^ Major surgery increases the risk of fatal events during hospital stay and after discharge from hospital.^2^ For years, anesthesiologists have been debating whether the type of anesthetic technique can decrease mortality during the follow-up period.^2^

Anesthesia can be divided into two major techniques: general anesthesia and neuraxial anesthesia.^2^ Anesthetic procedures in which patients are subjected to central neurological depression using gaseous or intravenous drugs are called general anesthesia, but situations in which a local anesthetic agent is used next to the spinal cord is termed neuraxial anesthesia.^2^ In this second group, when the injection is into the subarachnoid space, it is called spinal anesthesia, and when it is into the epidural space, it is called epidural anesthesia.^2^ Neuraxial anesthesia has some physiological effects that seem less invasive than general anesthesia and which may improve the outcome.^1^^,^^2^

A systematic review of randomized controlled trials (RCTs) showed that neuraxial anesthesia can decrease postoperative mortality consequent to abdominal surgery by 30%, with postoperative analgesia for 24 hours, in comparison with general anesthesia.^2^ This result can be criticized because it cannot be extended to all types of surgery in clinical practice_._^1^^,^^2^ A systematic review relating to urological surgery analyzed pain scores and other secondary outcomes without looking for mortality in the context of the choice of anesthetic technique.^1^^,^^3^ In this context, we conducted a study to answer one research question: what is the difference in mortality rate between using general anesthesia and using neuraxial anesthesia for urological surgery?

## OBJECTIVE

The purpose of this systematic review was to compare the effectiveness and safety of neuraxial anesthesia versus general anesthesia for urological surgery.

## METHODS

### Protocol

A protocol was initially developed, and this is available from the corresponding author on request. This systematic review was carried out using methods established by the Cochrane Collaboration.^4^ We used scientific methods to analyze published papers rather than patients, without correlating our results with specific journals, patients or institutions. Thus, the present research was not presented to any ethics committee. We followed the items for systematic reviews and meta-analyses presented in the PRISMA (Preferred Reporting Items for Systematic Reviews and Meta-Analyses) statement.^5^

### Eligibility criteria

*Types of participants:* The patients included in this review were 18 years of age or older, with urological disorders, and were treated surgically. Patients who underwent urological surgery performed together with other types of surgery were excluded.

*Types of studies:* Only randomized controlled trials (RCT) were included in this systematic review. Data from studies published twice were gathered from the study with the best description. Studies with incomplete data descriptions were excluded.

*Types of interventions:* The intervention group was neuraxial anesthesia. The control group was general anesthesia. Catheter use in neuraxial anesthesia techniques was not an exclusion criterion.

### Identification of studies

The following databases were searched: Cochrane Central Register of Controlled Trials (CENTRAL) in the Cochrane Library (Issue 10, 2012); Medline (Medical Analysis and Retrieval System Online), via PubMed (1966 to October 2012); Lilacs (Literatura Latino-Americana e do Caribe em Ciências da Saúde), available at http://regional.bvsalud.org/php/index.php (1982 to October 2012); SciELO (Scientific Electronic Library Online), available at http://www.scielo.br (the last search was in October 2012); and Embase (Excerpta Medica Database), available at http://aplicacao.periodicos.saude.gov.br/ (1974 to October 2012). The reference lists of the studies included, and the reference list of one systematic review in this field that was published before the present study were also searched.^3^ There were no restrictions on any language, date or document format.

The search strategy used in Medline via PubMed was adapted and used for CENTRAL. We used the terms anesthesia and urological surgeries for Lilacs. We used the terms anesthesia and urology for SciELO. The search strategy for EMBASE was ‘general anesthesia’/exp OR ‘spinal anesthesia’/exp OR ‘epidural anesthesia’/exp AND rand* AND ‘Urologic Surgical Procedures’/exp [embase]/lim. The search strategy for PubMed can be seen in Table 1.

**Table 1.** Search strategies for Medline via PubMed

Database	Search strategy
PubMed	(“anesthesia, general”[MeSH Terms] OR “anesthesia, inhalation”[MeSH Terms] OR “anesthesia, intravenous”[MeSH Terms] AND “anesthesia, conduction”[MeSH Terms] OR “anesthesia, epidural”[MeSH Terms] OR “anesthesia, spinal”[MeSH Terms])
	AND
	(“urologic surgical procedures”[MeSH Terms] OR urologic surgery[Text Word])
	AND
	(randomized controlled trial [Publication Type] OR controlled clinical trial [Publication Type] OR randomized controlled trials [MeSH Terms] OR random allocation [MeSH Terms] OR double blind method [MeSH Terms] OR single blind method [MeSH Terms] OR clinical trial [Publication Type] OR clinical trials [MeSH Terms] OR (clinical* [Text Word] AND trial* [Text Word]) OR single* [Text Word] OR double* [Text Word] OR treble* [Text Word] OR triple* [Text Word] OR placebos [MeSH Terms] OR placebo* [Text Word] OR random* [Text Word] OR research design [MeSH Terms] OR comparative study [MeSH Terms] OR evaluation studies [MeSH Terms] OR follow-up studies [MeSH Terms] OR prospective studies [MeSH Terms] OR control* [Text Word] OR prospectiv* [Text Word] OR volunteer* [Text Word])

### Selection of studies

Titles, abstracts, or both, identified by the search strategy for PubMed and other databases, were independently reviewed by two investigators (FTB and AAC). Subsequently, RCTs that were identified as potentially providing answers for our research question were requested so that the full text could be read. Data from the RCTs were recorded on a standardized form developed by the authors. Discordances were resolved by means of consensus meetings.

### Assessment of methodological quality and risk of bias

The study validity of the RCTs was investigated by two authors independently, using several scales: the Jadad score; the risk of bias table (Rob table) suggested by the Cochrane Handbook; and the Delphi List.^4^^,^^6^^,^^7^

The Jadad score was based on three items.^6^ The first item was given one point when the randomization was cited; another point was added if the randomization method was described and appropriate; and one point was deducted if this step was described incorrectly. The second item was given one point when the study was double-blind; another point was added if this method was described and appropriate; and one point was deducted if this step was described incorrectly. The third item was given one point when the numbers and reasons for withdrawals and dropouts were reported. Trials scoring three or more points were considered as having good validity.

The Rob table analyzes the following:^4^ sequence generation, allocation sequence concealment, blinding, incomplete outcome data, selective outcome reporting and other sources of bias. Each item was judged subjectively, looking for bias. Three categories were possible: low risk of bias, high risk of bias, or unclear risk of bias.

The Delphi List consists of several questions:^7^ 1. “Was a randomization method used?” 2. “Was the treatment allocation concealed?” 3. “Were the groups similar at baseline regarding the most important prognostic indicators?” 4. “Were the eligibility criteria specified?” 5. “Was the outcome assessor blinded?” 6. “Was the care provider blinded?” 7. “Was the patient blinded?” 8. “Were point estimates and variability measurements presented for the primary outcome?” and 9. “Did the analysis include an intention-to-treat analysis?” The answers could be yes, no, or “don’t know”.

### Outcomes

The primary outcome was mortality. Mortality was defined as a fatal event during surgery or within the first year afterwards.^2^

The secondary outcomes were: stroke, myocardial infarction, length of hospitalization, quality of life, degree of satisfaction, postoperative cognitive dysfunction and blood transfusion requirements. Stroke was loss of brain function caused by a disturbance in brain blood supply. Myocardial infarction was loss of cardiac function caused by a disturbance in coronary blood supply.^2^ Length of hospitalization was duration of hospital stay. Quality of life was the aspect of life that was influenced by physical wellbeing or mental status_._^4^ Degree of satisfaction was the patient’s reaction to the healthcare received.^8^ Postoperative cognitive dysfunction was a state of mental confusion after surgery. Blood transfusion requirement was considered to be the number of blood units transfused.

Internal validity, external validity and statistical treatment were analyzed. Internal validity was the possibility that the results could be applied to other patients in clinical practice.^9^ External validity was the concept of conducting studies with the minimum possibility of bias.^9^ Statistical treatment was considered to be the hypothesis tests used.

### Data analysis

It was planned to perform the meta-analyses using the Review Manager statistics. For dichotomous outcomes, the relative risk and number needed to treat were calculated with 95% confidence intervals using a random-effect model (REM), and for continuous outcomes, the weighted mean difference was calculated with its 95% confidence interval using a random-effect model. Statistical heterogeneity was assessed by using heterogeneity tests: standard chi-square test and the I-square test, such that I^2^ > 50% implied significant heterogeneity.^10^ The concordance between the authors was analyzed using the kappa statistic coefficient. We analyzed clinical and methodological heterogeneity by comparing the methodology used, characteristics of the participants included, and types of intervention in the eligible articles. We used simple frequencies for all outcomes.

## RESULTS

### Study selection

**Figure 1.**
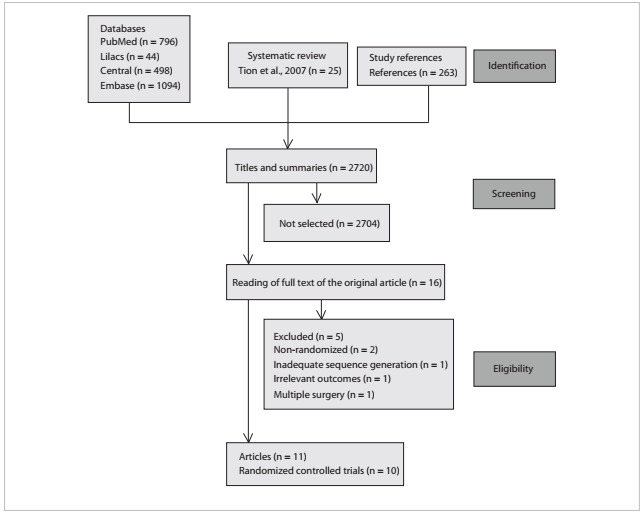
Trial flow.

Figure 1 demonstrates the process used for selecting relevant articles. We identified 2720 articles from running the search strategy, which led to identifying 16 papers for further analysis. We did not identify any titles in SciELO. In the selection process, five articles were subsequently excluded. The reasons for exclusion can be seen in Figure 1. The authors found that 11 articles had the potential to answer our research question,^11^^,^^12^^,^^13^^,^^14^^,^^15^^,^^16^^,^^17^^,^^18^^,^^19^^,^^20^^,^^21^ but one RCT had been published twice, and data were gathered from the best description.^18^^,^^19^ The kappa statistical coefficient was 0.8.

### Study validity

**Figure 2.**
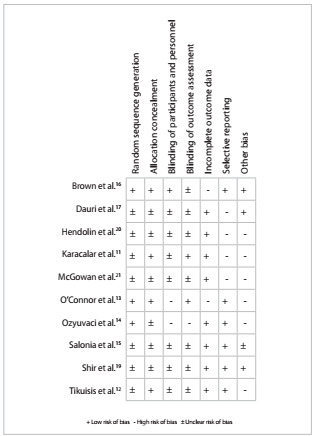
Risk of bias summary for each study included.

The analysis on study validity demonstrated the following: one of these 10 RCTs presented a Jadad score greater than three;^16^ two RCTs had more than five “yes” answers in the Delphi list analysis;^13^^,^^16^ the allocation concealment was described correctly in four RCTs;^11^^,^^13^^,^^14^^,^^16^ the sequence generation method was not described in seven RCTs;^11^^,^^12^^,^^15^^,^^17^^,^^19^^,^^20^^,^^21^ and blinding was not described in eight RCTs.^11^^,^^12^^,^^13^^,^^14^^,^^15^^,^^17^^,^^19^^,^^21^ One study was described as single-blind.^13^ The length of follow-up was three months in one RCT whereas it was the length of hospital stay in the other RCTs.^19^ The risk-of-bias summary for each study included can be seen in Figure 2.

### Outcomes

The characteristics of the selected RCTs analyzed and their outcomes are in Table 2.^11^^,^^12^^,^^13^^,^^14^^,^^15^^,^^16^^,^^17^^,^^18^^,^^19^^,^^20^^,^^21^ Pooling the results to produce a meta-analysis was not possible. The decision not to perform a meta-analysis took into account the fact that the interventions and outcomes assessed were different among the RCTs included. The reasons for not performing a meta-analysis in relation to each outcome are listed in the following paragraphs.

**Table 2.** Characteristics of the randomized controlled trials that compared neuraxial anesthesia and general anesthesia for urological surgery

Study (year)	Anesthesia	n	Type of surgery	% Male	Outcome	P value	Remarks
Karakalar et al.^11^	CSE GA	86 90	PNL	47.8 47.7	Satisfaction degree: CSE had better patient satisfaction.	0.001	Some patients received blood transfusion before surgery.
Tikuisis et al.^12^	EA + GA GA	27 27	RP	100 100	Blood transfusion requirements: less blood was transfused under EA + GA.	0.007	Duration of surgery under EA + GA was lower.
O’Connor et al.^13^	EA + GA GA	49 50	RP	100 100	1. Myocardial infarction: EA + GA showed one episode of ST segment depression. 2. Length of hospitalization. 3. Blood transfusion requirements: number of patients transfused under EA + GA was lower.	1. NS 2. NS 3. 0.028	Duration of surgery under EA + GA was lower. Controlled hypotension was used only under EA + GA.
Ozyuvaci et al.^14^	EA + GA GA	25 25	RC	100 100	Blood transfusion requirements: more units of blood transfusion were used in GA group.	< 0.01	The anesthesiologists were free to use drugs and doses under general anesthesia.
Salonia et al.^15^	GA SA	36 34	RRP	100 100	Blood transfusion requirements: overall blood loss was less under SA.	0.04	Intraoperative autologous and homologous transfusions were used.
Brown et al.^16^	SA + GA GA	49 50	RP	100 100	1. Mortality, myocardial infarction and length of hospitalization: were reported as ‘other perioperative outcomes’. 2. Length of hospitalization: GA had more time. 3. Quality of life: eight subscales and two composite scores of the SF-36 were used.	1. N/A 2. 0.01 3. NS	There was no mortality or myocardial infarction data in results section. Quality of life in the study population was better than U.S. population.
Dauri et al.^17^	EA + GA GA	11 9	RT	53.8 58.3	Length of hospitalization: EA + GA had lower mean.	N/A	Demographic data were not reported.
Shir et al.^19^	EA EA + GA GA	33 34 33	RRP	100 100 100	1. Mortality: no deaths for three months. 2. Stroke: no neurological complications for three months. 3. Myocardial infarction: Length of hospitalization: median was similar. 4. Blood transfusion requirements: less blood transfusion was performed under EA during surgery.	1. N/A 2. N/A 3. 0.12 4. 0.02	This study was published twice.^17,18^
Hendolin et al.^20^	EA GA	17 21	RRP	100 100	Blood transfusion requirements: five patients under GA and one under EA received blood transfusion.	N/A	Correlation test was used but was not reported in ‘material and methods’.
McGowan et al.^21^	SA GASV GACV	50 50 50	TP	100 100	1. Mortality: four patients died (2.6%). 2. Myocardial infarction: one patient in GACV group. 3. Length of hospitalization: means were statistically the same. 4. Blood transfusion requirements: number of patients transfused was greater in GACV group.	1. N/A 2. N/A 3. >0.05 4. N/A	Surgical procedures were performed by two urologists. GSCV group had more patients transfused but had the biggest prostate between the three groups.

GA = general anesthesia; SA = spinal anesthesia; EA = epidural anesthesia; CSE = combined spinal epidural anesthesia; GASV = general anesthesia with spontaneous ventilation; GACV = general anesthesia with controlled ventilation; PNL = percutaneous nephrolithotripsy; RP = radical prostatectomy; RC = radical cystectomy; RRP = Radical retropubic prostatectomy; TP = transurethral prostatectomy; RT = renal transplantation; NS = not significant; N/A = not available; n = number of participants.

Mortality: Brown et al.^16^ reported intraoperative outcomes, while the data from Shir et al.^19^ and McGowan et al.^21^ were not taken into consideration because the anesthetic technique was not the same as used today. Shir et al.^19^ used sodium thiopental, succinylcholine, isoflurane and pancuronium bromide for general surgery. McGowan et al.^21^ used cinchocaine for neuraxial anesthesia and so-dium thiopental, succinylcholine and halothane for general surgery.

Stroke: only Shir et al.^19^ reported this outcome.

Myocardial infarction: O’Connor et al.^13^ reported that there were no cases among the groups. Brown et al.^16^ reported that one patient who presented bradycardia was withdrawn from the study to investigate myocardial infarction, which was not confirmed at the end of the study. The data from Shir et al.^19^ and McGowan et al.^21^ were not taken into consideration because the anesthetic technique used was not the same as used today.

*Length of hospitalization*: O’Connor et al.^13^ reported on patients whose hospital stay was five days or more. Brown et al.^16^ reported data correctly; Dauri et al.^17^ reported patients for renal transplantation; and the data from Shir et al.^19^ and McGowan et al_._^21^ were not taken into consideration because the anesthetic technique used was not the same as used today.

*Quality of life*: only Brown et al.^16^ reported this outcome.

*Degree of satisfaction*: only Karacalar et al.^11^ reported this outcome.

*Postoperative cognitive dysfunction*: none of the studies reported this outcome.

*Blood transfusion requirements*: Tikuisis et al.^12^ reported the mean without standard deviation; O’Connor et al.^13^ reported the number of patients who received blood transfusion; Ozyuvaci et al.^14^ reported this outcome for radical cystectomy; Salonia et al.^15^ reported autologous and heterologous blood transfusions; and the data from Shir et al.,^18^ Hendolin et al.^20^ and McGowan et al.^21^ were not taken into consideration because the anesthetic technique used was not the same as used today. Hendolin et al.^20^ used sodium thiopental, succinylcholine and alcuronium.The basilic vein was used for venous pressure measurement, and the left radial artery was cannulated for blood gas measurement.

## DISCUSSION

Systematic review is a research method that pools the results from individual trials and can resolve conflicts in the literature.^22^ However, the difference in mortality rate between neuraxial anesthesia and general anesthesia for urological surgery was not determined in the present study. Although 10 RCTs with 856 patients were identified, inadequate reporting of the internal validity topics (allocation, blinding, withdrawal and dropouts), presence of clinical heterogeneity (type of surgery, length of follow-up and presence of cancer) and drugs used in some studies that are not used today were limiting factors in this investigation. Before starting this study, we searched for systematic reviews and did not find any that analyzed mortality relating to the choice of anesthetic technique in urological surgery.^1^

The analysis on study validity showed that the reporting of internal validity and external validity items was not done well in the studies included. Only one study presented a Jadad score of more than 3; seven out of the 10 studies had the answer “unclear risk of bias” to most of the questions analyzed in the Rob table; and five studies did not fulfill at least half of the items in the Delphi list. According to the Cochrane Collaboration’s tool for assessing the risk of bias, the majority of the studies were generally poor. Random sequence generation, allocation concealment and blinding were problematic and were not well described or were not conducted adequately. Pooling the results from these studies would therefore produce doubts in this systematic review and the results would be questionable. Appropriate reporting of the methodological criteria for designing and conducting studies is important for ensuring quality and for making it possible to pool the results from the studies included in systematic reviews.^4^

A previous study showed that there was lower mortality in the neuraxial group than in the general anesthesia group, but that study was an analysis on patients who underwent abdominal surgery.^2^ We cannot demonstrate the same result as found in this previous study. Mortality was analyzed in three RCTs,^16^^,^^19^^,^^21^ but was not reported in one of these studies,^16^ and was not statistically significant different in the other two.^19^^,^^21^ One study reported mortality as ‘other outcomes’ and readers had to imagine that the frequency of this outcome was the same between the groups.^16^ Patients analyzed in RCTs should be followed up for more than three months, but this only occurred in one study.^19^ They should also have the same surgical procedure, and the personnel who are responsible for the data and for patient care have to be blinded to ensure homogeneity between the studies.

Stroke was analyzed in one RCT, with no differences between the groups.^19^ Demographic data has to be analyzed to show that patients have the same clinical conditions. The American Society of Anesthesiologists (ASA) classification shows physical status and analyzes the presence of diseases and medications, but comorbidities cannot be compared between groups using only the ASA classification. Thus, further information about groups is needed in order to pool the results in a meta-analysis. More RCTs are needed in order to answer our research question.

Epidural analgesia can provide cardioprotective effects, but one meta-analysis failed to show that this had any positive influences in non-cardiac surgery patients.^23^ Likewise, we were unable to show such results in urological surgery. Myocardial infarction was reported in four studies.^13^^,^^16^^,^^19^^,^^21^ Three studies did not report any data on this outcome,^16^^,^^19^^,^^21^ and in one study, the patients were not followed up for an adequate length of time and the authors did not analyze the data on the withdrawn patients because they had bradycardia and ST segment depression.^13^ Postoperative analgesia can enable lower myocardial exertion and provide cardioprotective effects, but this outcome remains doubtful.^23^ Moreover, we did not have data homogeneity that would allow pooling of the results from urological surgery so as to contest or confirm this result.

Length of hospitalization was reported in four RCTs.^13^^,^^16^^,^^17^^,^^21^ Two RCTs showed no differences between the groups,^13^^,^^21^ one RCT did not report any data,^17^ and one RCT demonstrated favorable results for neuraxial anesthesia.^16^ There was a tendency to consider that the hospital stay was decreased when neuraxial analgesia was used, but differences in clinical scenarios and in the format of the papers making the reports gave rise to heterogeneity. Patients should receive the same anesthetic techniques and authors should take adequate numbers of patients into account in order to have statistical power. This tendency needs to be proved in future RCTs, so as to change clinical practices.

Quality of life was reported in one RCT and no differences were seen.^16^ Myles et al.^24^ analyzed patients after cardiac surgery and showed that poor quality recovery may be predictive of poor quality of life until three months after surgery. RCT authors should provide more data about this outcome so that strategies can become more effective for improving the quality of care during surgery and hospital stay. For this purpose, it is advisable to use the same instrument to analyze the data. The best instrument should analyze the hospital stay and length of follow-up.

The degree of satisfaction was reported in one RCT and the spinal-epidural group had better patient satisfaction.^11^ The main causes of dissatisfaction were nausea, vomiting and postoperative pain. Reports on complications can help to create strategies for safety procedures, but if the different anesthetic techniques produce the same complications, patient satisfaction data can provide strategies for ensuring good quality of anesthesia administration.

Postoperative cognitive dysfunction was not reported. This outcome has now been correlated not only with general anesthesia but also with sedation for noninvasive procedures, cardiac surgery and non-cardiac surgery. The presence of delirium during hospital stay carries a high mortality risk, particularly in older patients.^25^^,^^26^ RCT authors can provide data and analyze this outcome several years after surgery to investigate differences in mortality data.

Transfusion requirements were reported in seven RCTs.^12^^,^^13^^,^^14^^,^^15^^,^^19^^,^^20^^,^^21^ Three RCTs showed favorable results when general anesthesia and epidural anesthesia was used together;^12^^,^^13^^,^^14^ two RCTs demonstrated favorable results for neuraxial anesthesia;^15^^,^^19^ and data were unavailable in two RCTs.^20^^,^^21^ There was a tendency to consider that blood transfusion requirements were lower when neuraxial anesthesia was used, but confounding factors may have been contributing towards this result in the studies included. It is well known that anesthesia with controlled hypotension can reduce blood loss, and therefore the transfusion requirements may become lower because of the anesthetic techniques. However, different surgical strategies may produce the same result. RCT authors should provide data about prostatic gland weight, antifibrinolytic therapy, controlled hypotension techniques, patients’ ages and surgical techniques, so that it becomes possible to pool the results. This tendency has to be proved in future RCTs, so as to change clinical practices.

In future research, it will be necessary to pay attention to mortality and other outcomes that may provide answers regarding which anesthetic technique is best for urological surgery. The factors involved may include stroke, myocardial infarction, length of hospitalization, quality of life, degree of satisfaction, postoperative cognitive dysfunction and blood transfusion requirements. These topics were not taken into consideration in all the RCTs included in this study, and the length of follow-up only reached as much as three months in one RCT. By making the assumptions of 5% mortality in the general anesthesia group, 1% mortality in the neuraxial anesthesia group, 80% power and 5% significance level, 284 participants will be necessary in each group, for future studies to answer this research question. More RCTs with adequate numbers of patients and external and internal validity are needed.

The implications for clinical practice are that so far, it is not possible to say which anesthetic technique is better for urological surgery, between neuraxial anesthesia and general anesthesia, taking the factors of mortality, stroke, myocardial infarction, length of hospitalization, quality of life, degree of satisfaction, postoperative cognitive dysfunction and blood transfusion requirements into account. More RCTs are needed for analyzing patients with urological diseases, with adequate internal validity and length of follow-up. Finding an anesthetic technique that has lower mortality and better other outcomes can help in deciding which anesthetic technique is the best, and this aim should be considered in all future RCTs. It is important to take efficiency and safety into consideration at the time of choosing the anesthetic technique, and so professionals should consider each patient and his or her comorbidities individually, in conjunction with their own clinical practice, professional experiences and hospital work conditions at the time of the urological surgery. Each patient should be analyzed individually at the time of choosing the anesthetic technique.

## CONCLUSION

At the moment, the scientific evidence available cannot prove that neuraxial anesthesia is more effective and safer than general anesthesia for urological surgery. There were insufficient data to pool the results relating to mortality, stroke, myocardial in-farction, length of hospitalization, quality of life, degree of satisfaction, postoperative cognitive dysfunction and blood transfusion requirements.
